# Exploring Polygenic Risk and Neuroimaging Parameters across the Alzheimer's disease Spectrum

**DOI:** 10.1002/alz.084291

**Published:** 2025-01-09

**Authors:** Snehal Pandya, Annalena Venneri, Matteo De Marco

**Affiliations:** ^1^ Brunel University London, London UK; ^2^ Brunel University London, Uxbridge, UB8 3PH UK

## Abstract

**Background:**

Sporadic Alzheimer’s disease (AD) accounts for >90% of AD cases, of which 70% are thought to be due to a combination of several risk genes. Of these, Apolipoprotein E (APOE) is the most studied gene. Given that the APOE ɛ4 risk variant is found in ∼14% of the general population and ∼37% of the AD population, APOE ɛ4 is neither necessary nor sufficient to cause AD on its own. Investigating polygenic scores may lead to a better predictive model of AD, by studying many genes simultaneously, and increase understanding of how gene‐gene interactions may contribute to AD. The aim of this research was to review systematically papers exploring polygenic risk and neuroimaging parameters across the AD spectrum.

**Method:**

Literature searches were conducted on three online databases: PubMed, Scopus, and Web of Science, between January–March 2023. All articles and early access papers, written in English, and on humans, were included. Reviews and systematic reviews were excluded. Papers were cross‐checked by three independent reviewers. Selected papers were assessed with a customised set of criteria to determine quality papers for this review and to avoid biases.

**Result:**

3264 papers were found, of which 66 were eligible for review. Some papers used more than one imaging modality, therefore, there were a total of 80 studies: 49 structural, 8 functional, and 23 neuromolecular, covering regional and whole‐brain metrics. Polygenic risk scores and polygenic hazard scores are associated with regions vulnerable to AD, e.g., hippocampi, and entorhinal cortices. These findings are consistent across the three imaging modalities, and across the AD spectrum. In some cases, significant associations between polygenic scores and neuroimaging parameters are only present when including, or excluding, APOE. There are discrepancies in whether APOE increases, or decreases, strength of associations.

**Conclusion:**

This review demonstrates that attention must divert away from APOE since numerous genes impact brain architecture and connectivity, and it is their combined effect that is of key interest in sporadic AD. It highlights the challenges faced, and the intricacies that must be thought about, when constructing polygenic scores.

